# Urban energy exchanges monitoring from space

**DOI:** 10.1038/s41598-018-29873-x

**Published:** 2018-07-31

**Authors:** Nektarios Chrysoulakis, Sue Grimmond, Christian Feigenwinter, Fredrik Lindberg, Jean-Philippe Gastellu-Etchegorry, Mattia Marconcini, Zina Mitraka, Stavros Stagakis, Ben Crawford, Frans Olofson, Lucas Landier, William Morrison, Eberhard Parlow

**Affiliations:** 1 Foundation for Research and Technology Hellas, Institute of Applied and Computational Mathematics, Remote Sensing Lab, N. Plastira 100, Vassilika Vouton, 70013 Heraklion, Greece; 2 University of Reading, Reading, UK; 3 University of Basel, Basel, Switzerland; 4 University of Gothenburg, Gothenburg, Sweden; 5 Centre d’Etude Spatiale de la Biosphère (CESBIO), Toulouse, France; 6 German Aerospace Center (DLR), Oberpfaffenhofen, Germany

## Abstract

One important challenge facing the urbanization and global environmental change community is to understand the relation between urban form, energy use and carbon emissions. Missing from the current literature are scientific assessments that evaluate the impacts of different urban spatial units on energy fluxes; yet, this type of analysis is needed by urban planners, who recognize that local scale zoning affects energy consumption and local climate. Satellite-based estimation of urban energy fluxes at neighbourhood scale is still a challenge. Here we show the potential of the current satellite missions to retrieve urban energy budget fluxes, supported by meteorological observations and evaluated by direct flux measurements. We found an agreement within 5% between satellite and *in-situ* derived net all-wave radiation; and identified that wall facet fraction and urban materials type are the most important parameters for estimating heat storage of the urban canopy. The satellite approaches were found to underestimate measured turbulent heat fluxes, with sensible heat flux being most sensitive to surface temperature variation (−64.1, +69.3 W m^−2^ for ±2 K perturbation).  They also underestimate anthropogenic heat fluxes. However, reasonable spatial patterns are obtained for the latter allowing hot-spots to be identified, therefore supporting both urban planning and urban climate modelling.

## Introduction

**Figure 1:**
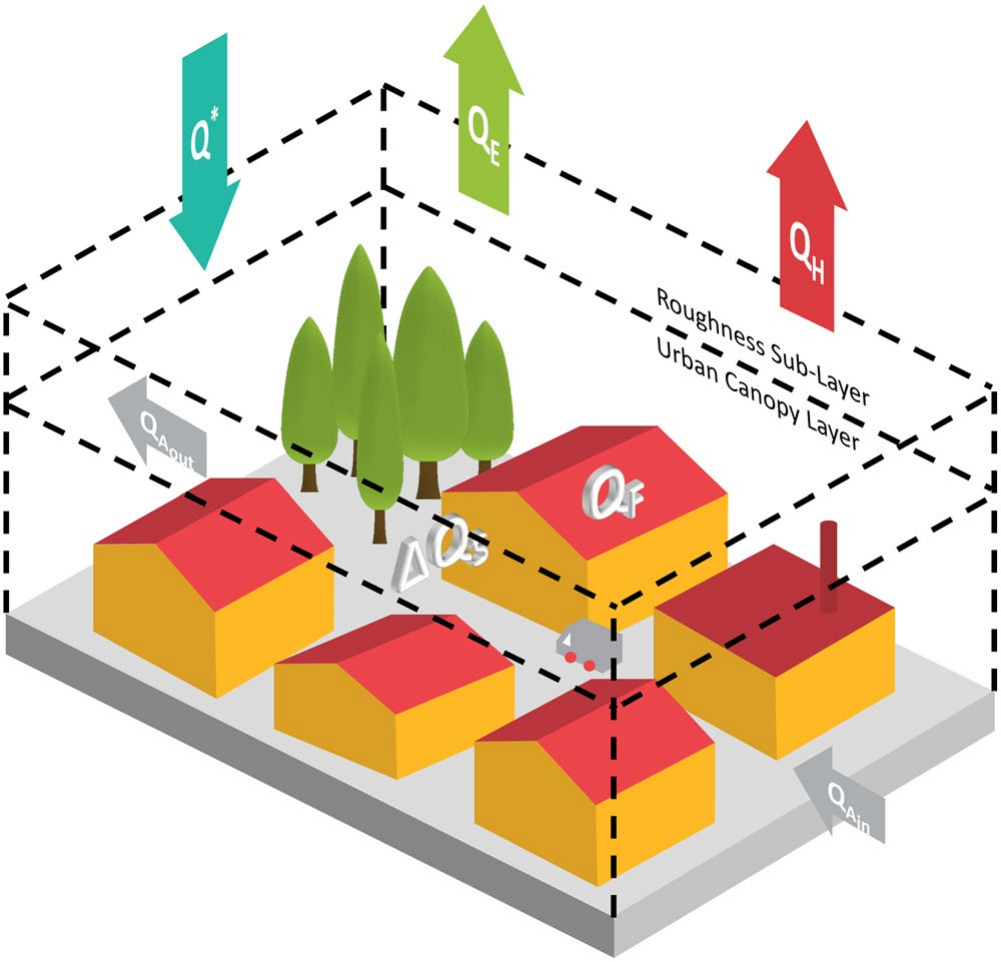
Conceptual illustration of the fluxes in the energy balance of an urban building–vegetation-soil–air volume. The equivalent surface energy budget per unit surface area through the top of the volume is: Q^*^ + Q_F_ = Q_H_ + Q_E_ + ΔQ_S_ + ΔQ_A_ (W m^−2^), where, Q^*^ is the net all-wave radiation flux; Q_F_ is the anthropogenic heat flux (resulting from vehicular emissions, heating and cooling of buildings, industrial processes and metabolic heat release by people and animals); Q_H_ is the turbulent sensible heat flux; Q_E_ is the turbulent latent heat flux; ΔQ_S_ is the net change in heat storage within the volume; and ΔQ_A_ is the net advected flux. Arrows are drawn in the direction to which the corresponding flux is considered positive. ΔQ_s_ and ΔQ_A_ (=Q_A,in_ − Q_A,out_) are positive if the internal energy of the volume increases.

The Urban Energy Balance (UEB) needs to account for the 3D nature of cities, quantifying the fluxes into, out of and the storage change within the control volume (Fig. 1). In the last 15 years, significant advances in understanding urban processes have benefited from enhanced computational capacity, improved resolution of satellite sensors and increased ability to couple advance urban surface parameterization schemes with atmospheric models^1–9^. Earth Observation (EO) has been widely used to study the Urban Heat Island (UHI) phenomenon, but to a lesser extent to quantify heat fluxes^10–15^. Recent studies^16,17^ have investigated the potential of EO to derive turbulent heat fluxes and identify and analyse the associated uncertainties.

Both Earth system science and urban planning communities need spatially disaggregated UEB data at local scale^18–20^ (neighbourhood, e.g., order (100 m × 100 m) or larger). However, such information is practically impossible to derive for extensive areas by *in-situ* flux measurements and EO-based estimation of spatio-temporal patterns of different UEB components is challenging. Therefore, the question arises whether EO can provide reliable estimates of UEB at the times of satellite acquisitions. Here we address this by investigating the potential of the current satellite missions to retrieve UEB fluxes at local scale, supported by meteorological observations. Recognising the range of city forms, size and settings, we explored three locations: London (UK), Basel (Switzerland) and Heraklion (Greece).

## Results and Discussion

**Figure 2:**
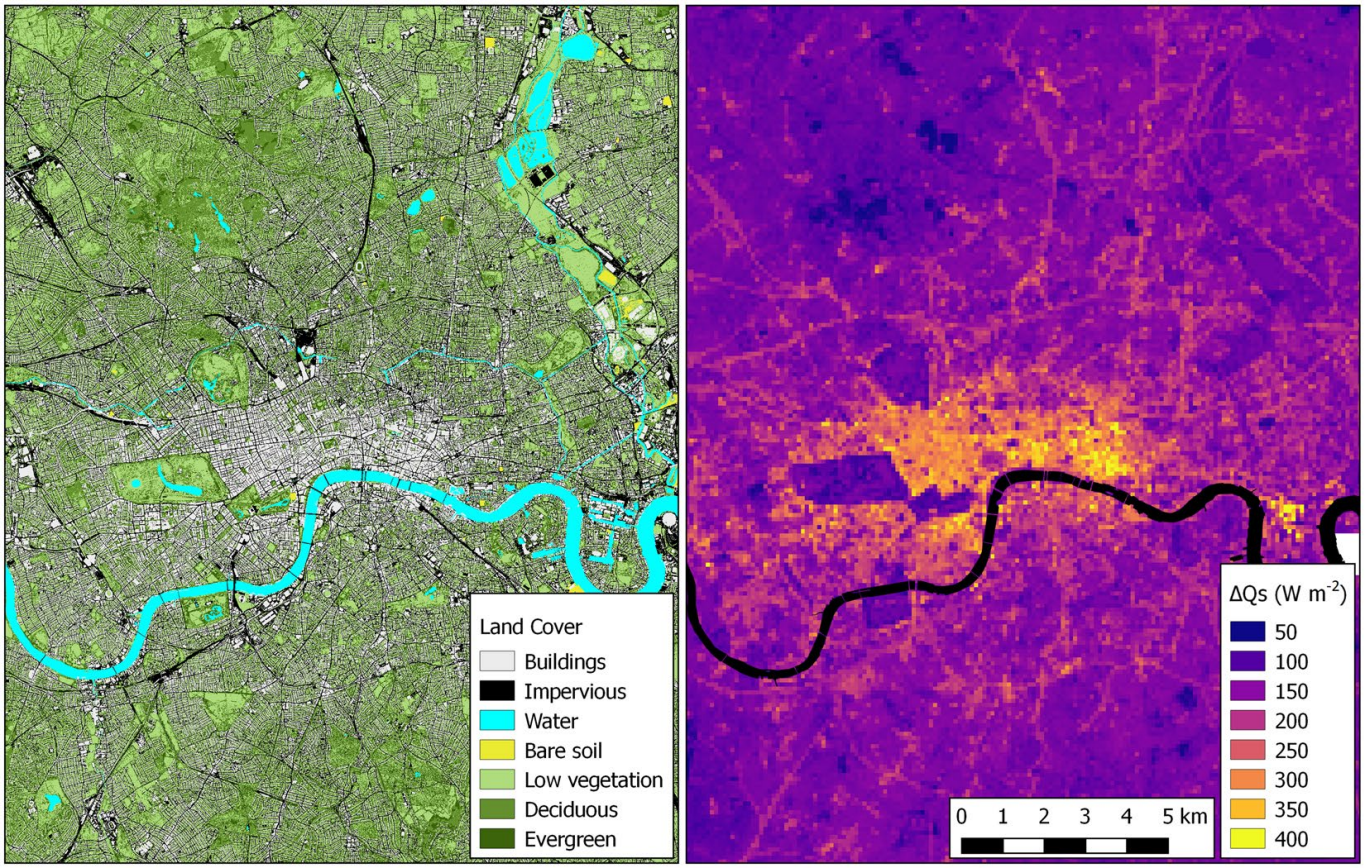
Land cover and heat storage for part of London, UK. (**a**) Land cover (seven classes: buildings, impervious surfaces (i.e., roads, parking lots, sidewalks, etc.), water, bare soil, low vegetation (<2 m) and high vegetation (≥2 m) split into evergreen and deciduous trees). (**b**) Net change in heat storage (ΔQs) (100 m × 100 m) on 19 July 2016 at 11:00 UTC. Highest values are observed in central densely built areas (City of London, Canary Warf (centre right)). River Thames is masked (black). Maps created with QGIS software, version 2.18 (www.qgis.org).

EO data from various sources were used to extract urban surface morphology and characteristics. Surface cover and material type were derived using advanced machine learning and fusion techniques and detailed spectral un-mixing approaches^21^ (e.g., Fig. 2a). Using EO-derived high resolution Digital Surface Models (DSM), surface roughness parameters (such as plan area index, frontal area index, roughness length and zero-displacement height) were calculated by morphometric analysis^22^. Examples of the morphometric analysis results are given in Supplementary Fig. S1. Satellite-derived thermal radiance at 1 km × 1 km was downscaled^23^ to 100 m × 100 m. Although uncertainties of the downscaling method exist, as Mitraka *et al*.^24^ noted, the error of downscaled temperatures, evaluated using *in-situ* surface temperature observations from the micrometeorological flux towers, is within 2.4 K (Supplementary Fig. S2).

The study period (January 2016–May 2017) allowed the seasonal cycle to be examined (Supplementary Fig. S3, London). For all cities this was appropriate with net all-wave radiation (Q*), net change in heat storage (ΔQ_S_) and turbulent sensible heat flux (Q_H_) peaking in summer. Q_H_ and ΔQ_S_ peak in August, whereas turbulent latent heat flux (Q_E_) is relatively minor throughout the year in city centres.

**Figure 3:**
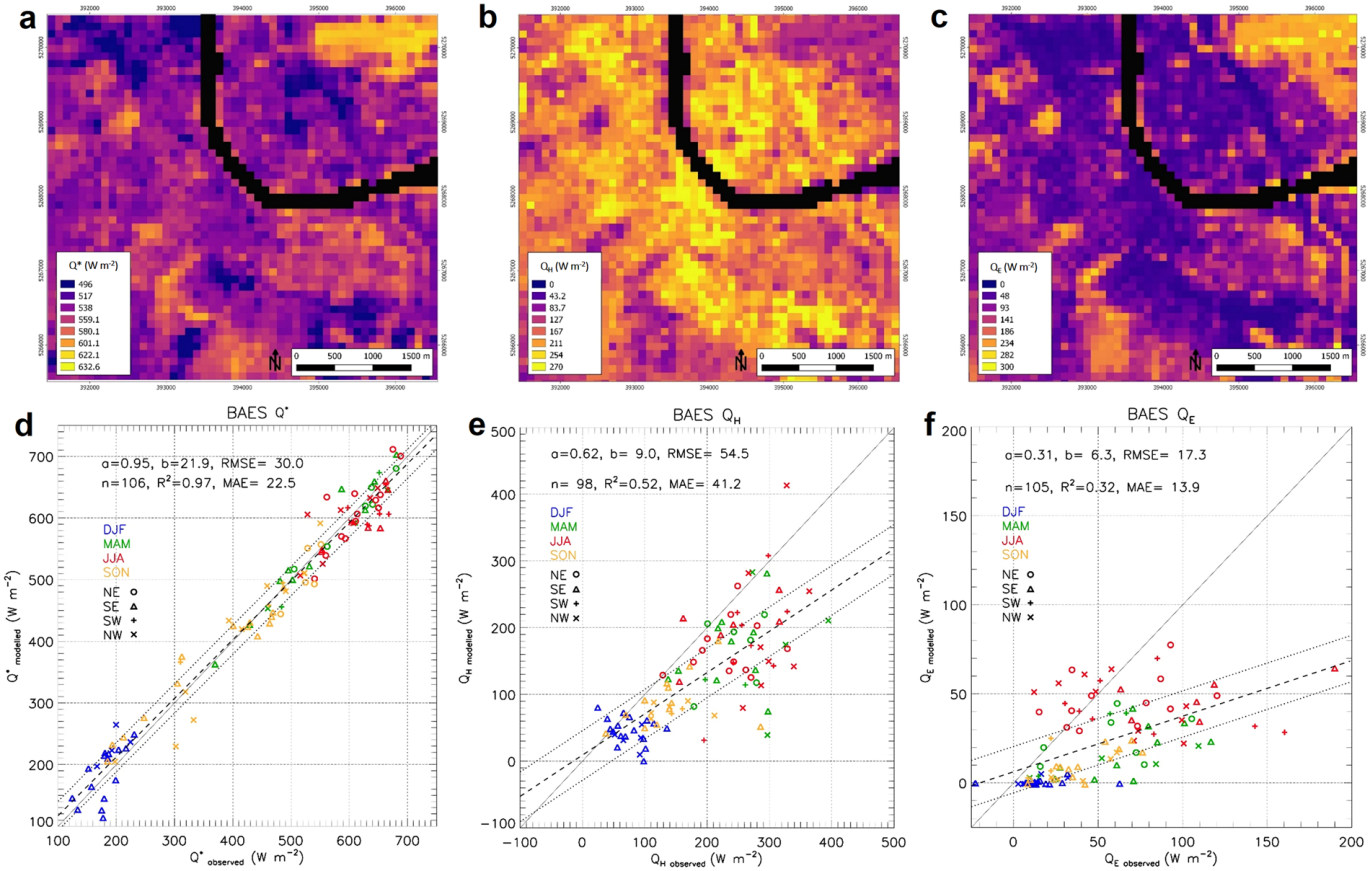
Spatial patterns of radiation and turbulent heat fluxes. (**a**–**c**) EO-derived fluxes for the central area of Basel (on 23 August 2016 at 09:50 UTC, grid reference UTM 32 N (EPSG:32632)); and (**d**–**f**) modelled versus observed fluxes at BAES (Basel Aeschenplatz) flux tower for all 2016 cloud-free satellite scenes. **(a**,**d)** net all wave radiation **(**Q*); (**b**,**e**) turbulent sensible heat flux (Q_H_); **(c**,**f**) turbulent latent heat flux (Q_E_). Comparison with observations are stratified by season (colours, *DJF refers to DEC, JAN, FEB, etc*.) and wind direction (symbols); 1:1 line (solid), linear regression best fit line (dashed) and extent of 80% of the data points (dotted lines) indicated. Linear regression statistics, a: slope, b: intercept (W m^−2^), R^2^: coefficient of determination, MAE: mean absolute error (W m^−2^), RMSE: root mean square error (W m^−2^). Maps created with QGIS software, version 2.18 (www.qgis.org). Water surfaces are masked.

Deriving Q* maps from EO is not straightforward as urban reflectance and thermal emission present anisotropic behaviour, caused by illumination geometry, 3D urban geometry and the distribution of urban material optical and thermal properties^2^. Here, the DART (Direct Anisotropic Radiative Transfer) model^6^ takes into account this anisotropy. It simulates reflected and emitted radiation using information derived from EO satellite images (see more details in Methods). For example, Q* in Basel (Fig. 3a) range between 496 and 633 W m^−2^. The highest fluxes occur near the river and in a vegetated region in the upper right of the domain. The mean absolute error (MAE, observed - modelled (EO-derived)) in Q* for 2016 was 22.5 W m^−2^ (Fig. 3d), with  agreement within 5%. This corresponds to an average root mean square error (RMSE) of 13 W m^−2^ and R² > 0.99 (Supplementary Fig. S4). Uncertainty arises from simplifying the complex urban surface structure and the surface elements (e.g., walls) unseen by satellites, except through multiple scattering, although they contribute to Q*.

ΔQ_S_ of an urban canopy is approximately 2–6 times larger than for non-urban canopies. Here, ΔQ_S_ is estimated using the element surface temperature method (ESTM)^3^, which reduces the 3D urban structure to one-dimensional (1D) elements (see details in Methods). The sensitivity analysis identified that the fraction of wall facet and materials are the two most important variables in the estimation of ΔQ_S_. Daytime peak (11:00 local time) ΔQ_S_ is largest (up to 400 W m^−2^) in densely built areas where tall buildings dominate (e.g., City of London and Canary Wharf in London Fig. 2b). Areas with low building density have, as expected, smaller ΔQ_S_ values. Uncertainty is assessed using two alternative approaches implemented within the SUEWS^7^ modelling platform: OHM^25^, and AnOHM^26^. All approaches produced similar results, but different spatial patterns are identified, mostly depending on the forcing data. To evaluate ESTM performance, observations from less complex sites with uniform land cover are used, where ΔQ_S_ can be directly measured with ground heat flux plates. MAE between 1.1 and 21.7 W m^−2^ is found.

The turbulent heat fluxes are strongly modified by large roughness elements and a complex mix of sources/sinks of heat and water in cities. Here, the Aerodynamic Resistance Method (ARM) is used to derive Q_H_^16,17^ and Q_E_^16^ at local scale. To implement this, EO-derived downscaled land surface temperature (LST) and surface roughness parameters are used combined with air temperature and humidity from meteorological stations (see details in Methods). For example, for 23 August 2016 in Basel (Fig. 3b), the highest Q_H_ values (up to 270 W m^−2^) are found in the most densely built-up and industrial areas. Q_H_ values have similar spatial patterns and magnitudes to ΔQ_S_, whereas, Q_E_ (Fig. 3c) is much smaller in the built-up areas (less than 150 W m^−2^) and has largest values in vegetated areas. The eddy covariance flux footprint^27^ is used to compare measured with modelled turbulent heat fluxes. For the Basel BAES (Aeschenplatz) flux tower, the Q_H_ MAE for 2016 is 41.2 W m^−2^ (Fig. 3e), with modelled Q_H_ underestimated compared to measured Q_H_. Similarly, the EO-derived Q_E_ is underestimated compared to flux tower observations (Fig. 3f). This relates to the uncertainties associated with ARM^16,17^ and the required input variables. Supplementary Figs S5 and S6 compare the EO-based turbulent sensible and latent heat fluxes with the ground truth at the three sites. A sensitivity analysis^17^ indicates that, overall, the EO-derived Q_H_ is most sensitive to LST variation (+69.3, −64.1 W m^−2^ for ±2 K perturbation).

**Figure 4:**
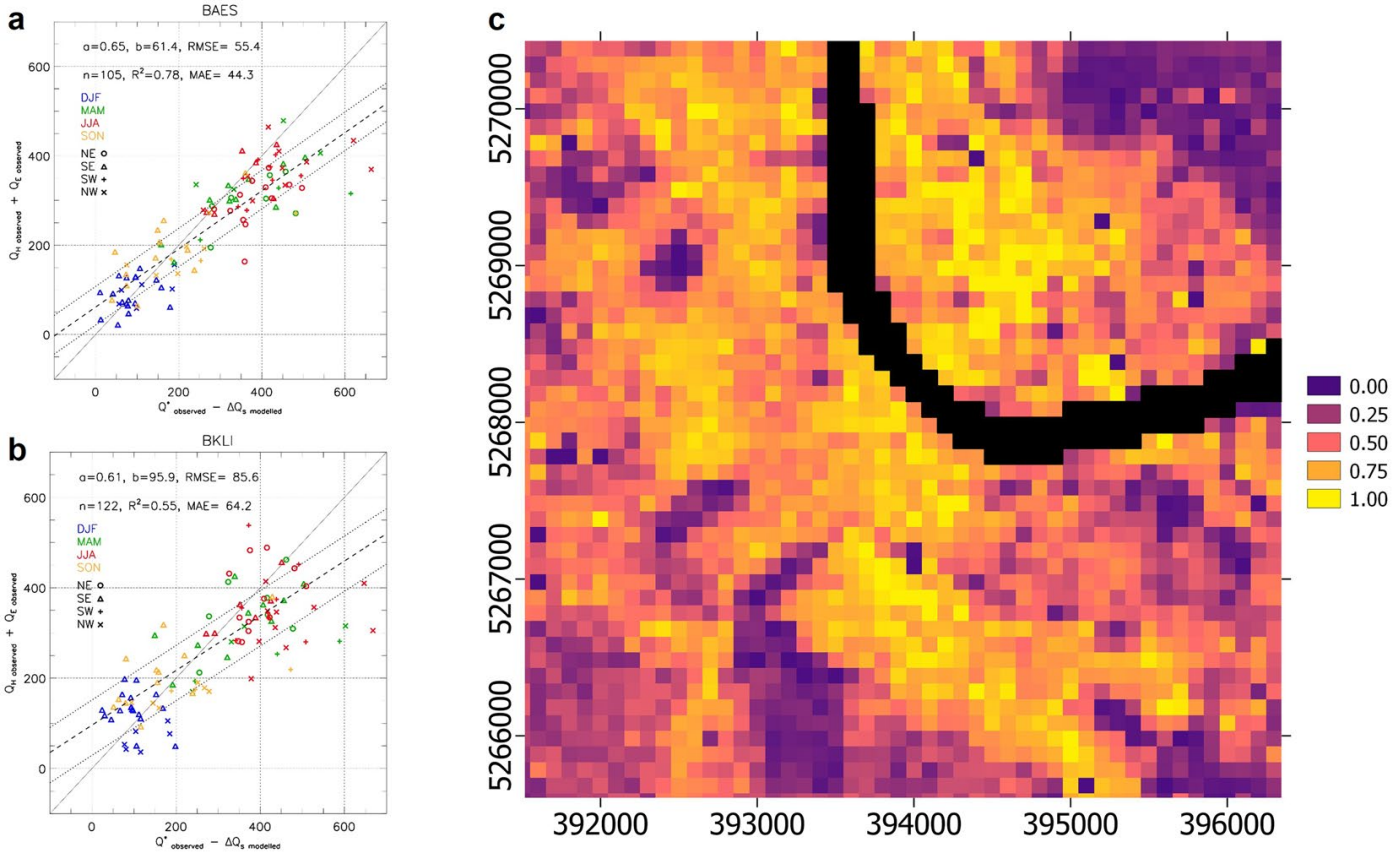
Anthropogenic heat flux (Q_F_) as a residual from regressing turbulent heat fluxes (Q_H_ + Q_E_) with available energy (observed Q* - modelled ΔQ_S_) in central Basel for all 2016 cloud-free satellite scenes stratified by season (colours, as Fig. 3d–f) and wind direction (symbols) for: (**a**) BAES (Basel Aeschenplatz (394330, 5267379)) and (**b**) BKLI (Basel Klingelbergstrasse (393221, 5268560)) flux towers, with 1:1 line (solid), linear regression best fit line (dashed) and extent of 80% of the data points (dotted lines). Linear regression statistics, a: slope, b: intercept (W m^−2^), R^2^, MAE (W m^−2^), RMSE (W m^−2^). (**c**) Relative spatial patterns of the anthropogenic heat flux (Q_F_) in central Basel for January and February 2016, scaled from 0 to 1 (unitless). The river is masked (black). Grid reference is UTM 32 N (EPSG:32632). Map created with QGIS software, version 2.18 (www.qgis.org).

Direct measurements of the anthropogenic heat flux Q_F_ are extremely difficult^28^. Here, Q_F_ is obtained from the residual of the UEB, and thus includes the cumulative uncertainties of all the terms. Energy balance closure typically compares available energy (Q* - ΔQ_s_) with turbulent heat fluxes (Q_H_ + Q_E_). Basel’s two flux towers, have total EC turbulent heat fluxes that tend to be slightly greater than the available energy during winter months (Fig. 4a,b), when Q_F_ is expected to be highest due to building space heating. The relative spatial pattern of EO-derived Q_F_ for central Basel (winter 2016, Fig. 4c) appears reasonable with clear correlation with building density. However, the absolute Q_F_ values for individual pixels include negatives (physically unrealistic). This suggests that individual UEB components are incorrect most likely due to the underestimation of Q_H_. Highest relative Q_F_ is found south of the river in the most densely built-up areas (Fig. 4c). Independent Q_F_ models based on inventories^29^ also confirm that buildings are the dominant source of Q_F_. This positive correlation between built-up areas indicates that EO can be used to identify hot-spots with relatively high Q_F_.

## Conclusions

Based on the analysis of individual UEB components, we conclude that current satellite missions have the potential to provide information about spatial patterns of urban energy exchanges, if supported by suitable meteorological measurements. Synergistic analysis of specific satellite observations is able to identify and map spatial distributions of heat fluxes at local scale, several times per day (if clear weather). The EO-based methods developed are easily transferable to any city and have the potential to support sustainable planning strategies, since knowledge of UEB patterns at neighbourhood level is needed in urban planning (e.g., to reduce or prevent Q_H_ and Q_F_ hot spots), health studies (e.g., to estimate impact on thermal comfort) and future proofing (e.g., to plan and implement interventions to reduce heat emissions). Finally, the frequency of heat waves is expected to increase^30^ with UHI and other urban characteristics exacerbating the respective warming, resulting in increased energy demand for cooling systems in low and mid-latitude cities, which in turn adds to heat emissions and raises temperatures further^31^. Our satellite-based approach, strengthened by uncertainty minimization in future applications, is expected to advance the current knowledge of the impacts of heat fluxes on energy consumption in cities, leading to the development of tools and strategies to mitigate these impacts, improving thermal comfort and energy efficiency.

## Methods

### Case studies and datasets

Three cities were selected: highly urbanized mega-city (London, UK); typical central European medium size city, that requires substantial winter heating (Basel, Switzerland); and a smaller, low latitude Mediterranean city that requires substantial summer cooling (Heraklion, Greece). Meteorological data from wireless automatic weather station networks (AWSN) and flux towers (net all-wave radiation and turbulent heat fluxes) in all cities were supplemented by Large Aperture Scintillometers^28^ in London. High spatial resolution satellite imagery (between 10 m × 10 m and 30 m × 30 m) from Copernicus Sentinel 2 and Landsat 5/7/8, as well as very high resolution (VHR) data (<10 m × 10 m) from Copernicus Contributing Missions, such as SPOT (Satellite Pour l’Observation de la Terre) and WorldView-2, RapidEye, TerraSAR-X and TanDEM-X are used. The EO thermal infrared data are from MODIS (Moderate Resolution Imaging Spectroradiometer), Sentinel 3 (1 km × 1 km) and ASTER (Advanced Spaceborne Thermal Emission and Reflection Radiometer) observations (at 90 m × 90 m)^21^. Synergies between Sentinels 2 and 3 proved useful to identify fractional surface cover needed in local scale surface emissivity and LST^23^ estimates. All UEB fluxes are estimated at 100 m × 100 m resolution.

### EO data analysis

Surface characteristics include morphology, cover and biophysical parameters. Morphology requires the location and dimensions of urban objects. For this purpose, DSMs are derived from VHR optical stereo imagery (Heraklion) or airborne LiDAR observations (Basel^21^ and London^32^). Surface structure parameters (i.e., building volume, sky view factor, plan area index, frontal area index, roughness length and zero-plane displacement height) are derived by morphometric analysis^22^, using the Urban Multi-scale Environmental Predictor (UMEP)^33^. Surface cover (plus its spatial and temporal changes) are obtained from EO using advanced machine learning techniques and detailed spectral mixture models^34^. Multi-temporal acquisitions are used to update the surface cover fraction maps for the classes of interest. By fusing VHR data with Sentinel-2 observations it is possible to categorize different roof materials^35^ and build spectral libraries^36^. Other required parameters (e.g., emissivity, albedo and vegetation indices) are derived as outlined in Marconcini *et al*.^21^. As daily high spatial resolution thermal imagery is unavailable, the thermal radiance retrieved at 1 km × 1 km resolution (MODIS, Sentinel 3) are downscaled to 100 m × 100 m LST and emissivity using Mitraka *et al*.’s^23^ novel technique. Uncertainties in this approach are quantified by Mitraka *et al*.^24^. The downscaled LST values are compared to *in-situ* surface temperature observations from the micrometeorological flux towers.

### Estimation of net all-wave radiation

Q* is the difference of the incoming solar (shortwave) radiation (K↓) and atmosphere thermal (longwave) emission (L↓), minus the outgoing reflected solar radiation (K↑) and urban surface thermal emission (L↑). Satellites observe in few spectral bands from restricted viewing directions, whereas Q* is an integral over a wide spectral domain and the whole hemisphere. Here, the DART model^9,37^ is used to estimate local scale Q*. DART simulates radiative transfer of the urban surface-atmosphere system. It operates on scenes simulated as 3D arrays of rectangular cells. Here a processing chain is developed that calibrates DART with satellite images: the iterative comparison of the model output with images allows the material optical properties (OP) per urban element at the spatial resolution of the satellite data to be obtained. The iterative procedure accounts for the multiple scattering mechanisms and has six steps: 1) Urban morphology and material type are given as input to DART. 2) DART simulates at high spatial resolution (e.g., 2.5 m) all spectral bands of the satellite sensor being used for comparison. The simulation accounts for the specific atmosphere and illumination conditions of each input satellite scene. In the first iteration, spatially constant OP are set per urban element type. 3) DART-simulated spectral image output are georeferenced and spatially resampled to the input satellite scene. 4) DART-simulated and satellite images are compared on a pixel basis to improve the OP map per urban element, using the area of the urban elements within each satellite pixel (derived from the urban morphology and material database). If the DART-simulated and satellite images do not match, the procedure re-iterates from step 2. 5) DART simulates the angular spectral radiative flux along all directions sampling the upper hemisphere. 6) Shortwave K* (K↓ - K↑) and longwave L* (L↓ - L↑) exitance maps are computed as a double integral over the spectrum and the upper hemisphere. Finally, Q* (K* + L*) is resampled to 100 m × 100 m spatial resolution and compared against observations from the micrometeorological towers.

### Estimation of the net change in heat storage

ΔQ_S_ is the net flow of heat stored in the urban volume (i.e., air, trees, buildings, ground, etc.). In urban areas, the net heat stored in the canopy is a relatively large fraction of Q* and directly evaluating ΔQ_S_ in the urban canopy is difficult^38^. To determine ΔQ_S_ we use the Element Surface Temperature Method (ESTM), which reduces the 3D urban volume to four elements, i.e., building roofs, walls, and internal mass and ground (road, vegetation, etc.)^6^:

1 $${\rm{\Delta }}{Q}_{S}={\sum }_{{\rm{i}}}\frac{{\rm{\Delta }}{T}_{i}}{{\rm{\Delta }}t}{\varrho }{c}_{i}{\rm{\Delta }}{x}_{i}\,{f}_{i},$$

where *ΔΤ*_*i*_*/Δt* is the rate of temperature change over the period for each element *i, ρc*_*i*_ is the volumetric heat capacity, *Δx*_*i*_ is the element thickness and *f*_*i*_ is the fraction of each surface type. Each element type has sublayers (e.g., a wall can be built up by brick, insulation and wood). Without internal element temperature data the average is determined:

2 $${\varrho }C\frac{\partial T}{\partial t}=-\,\frac{\partial Q}{\partial x}=-\,\frac{\partial }{\partial x}(-k\frac{\partial T}{\partial x}),$$

where *Q* is the conductive heat flux through the surface and *k* the thermal conductivity. For the internal surfaces (i.e., roof, exterior and interior walls, and floors) the surface temperature of element *i* is determined by setting the conductive heat transfer out of (in to) the surface equal to the radiative and convective heat losses (gains), as described by Offerle *et al*.^6^.

The morphology of the urban surface was derived from high resolution DSM, including 3D information of vegetation^32,33^. As land cover thermal properties differ, detailed land cover information was required. The seven land cover classes (Supplementary Fig. S1) have three surface materials types for impervious covers and five building categories. These are derived from Urban Atlas^39^ land use, street view photographs (ground level provided by Google Maps) and local knowledge. The meteorological forcing from the AWSN with the EO downscaled LST are used.

### Estimation of the turbulent heat fluxes

The methodology uses the ARM approach^40^ to estimate Q_H_:

3 $${Q}_{H}=\rho {c}_{p}\frac{LST-{T}_{a}}{{r}_{a}},$$

where ρ is the density of air, c_p_ the specific heat of air at constant pressure (1005 J kg^−1^ K^−1^), T_a_ is the air temperature provided by the AWSN and r_a_ is the aerodynamic resistance (s m^−1^). Analogously, Q_E_ is expressed as:

4 $${Q}_{E}=\frac{\rho {c}_{p}}{\gamma }\frac{{e}_{s}^{\ast }-{e}_{a}}{{r}_{a}+{r}_{s}},$$

where e_s_^*^ is the saturation water vapour pressure (hPa) at surface air temperature, e_a_ is the atmospheric water vapour pressure (hPa), *γ* is the psychrometric constant (0.67 hPa K^−1^) and r_s_ is the stomatal resistance (s m^−1^). Stomatal resistance is calculated following Kato *et al*.^41^ using a simplified equation from Nishida *et al*.^42^:

5 $$\frac{1}{{r}_{s}}=\frac{{f}_{1}({T}_{a}){f}_{2}(PAR)}{{r}_{sMIN}}+\frac{1}{{r}_{cuticle}},$$

where PAR is the photosynthetic active radiation, r_sMIN_ is the minimum stomatal resistance and r_cuticle_ is the canopy resistance related to the diffusion through the cuticle layer of leaves (10^5^ s m^−1^). Functions f_1_ and f_2_ are from Nishida *et al*.^42^ and r_sMIN_ is for each vegetation type^41^. Q_E_ is calculated by land cover type and weighted by the pervious r_sMIN_ type in every pixel. The aerodynamic resistance r_a_ is^40^:

6 $${r}_{a}=\frac{1}{{u}_{\ast }\kappa }[ln(\frac{{z}_{ref}-{z}_{d}}{{z}_{0m}})-{\psi }_{h}(\frac{{z}_{ref}-{z}_{d}}{L})+\,\mathrm{ln}(\frac{{z}_{0m}}{{z}_{0h}})],$$

with u_*_ the friction velocity (m s^−1^):

7 $${u}_{\ast }=U\kappa {[ln(\frac{{z}_{ref}-{z}_{d}}{{z}_{0m}})-{\psi }_{m}(\frac{{z}_{ref}-{z}_{d}}{L})-{\psi }_{m}(\frac{{z}_{0m}}{L})]}^{-1},$$

where *κ* is von K**á**rm**á**n’s constant (0.4), z_ref_ is height of wind measurements, z_d_ is the zero-plane displacement height, L is the Obukhov length, z_0m_ (z_0h_) is the roughness length and *ψ*_m_ (*ψ*_h_) is the stability function for momentum (heat). z_0h_ is often reported within the dimensionless number *kβ* ^*−*1^, defined as

8 $$\kappa {\beta }^{-1}=ln(\frac{{z}_{0m}}{{z}_{0h}}).$$

EC and scintillometry^28^ measurements can be used to determine *kβ* ^*−*1^ for their flux footprint. Here, flux tower measurements are used as reference values for the magnitude of the fluxes of momentum, sensible and latent heat during the satellite overpass. z_0h_ is calculated from the roughness Reynold’s number (Re_*_) as^43^:

9 $${z}_{0h}={z}_{0m}[7.4\,exp(-\alpha R{e}_{\ast }^{0.25})],$$

where *α* = 1.29 and Re_*_ (=*z*_*0m*_u_*_/*ν*) uses the kinematic molecular viscosity *ν* (1.461 × 10^−5^ m s^−1^).

The displacement height (*z*_*d*_) and *z*_*0m*_ are calculated with morphometic parameterization of Kanda *et al*.^44^, using urban characteristics (mean and maximum building height, standard deviation of building height, plan area index and frontal area index) derived from 1 m × 1 m DSM, using UMEP^22,33^.

To evaluate the EO-derived turbulent heat fluxes, EC observations are compared to modeled fluxes in the flux tower source area. Here, the Kormann and Meixner^27^ footprint model is used.

### Estimation of the anthropogenic heat flux

With the other UEB components known, Q_F_ is estimated as a residual and hence contains the net errors of all the components. The spatial Energy Balance Closure (EBC) can be determined from regression between (Q_H_ + Q_E_) and (Q^*^ - ΔQ_S_) to give an estimate of Q_F_ and uncertainty. This requires three assumptions: 1) All sources of anthropogenic heat are released into the environment. 2) Advection is negligible at the scale of interest or cancels between scales: at the microscale (e.g., Q_H_ between shadowed and sunlit patches, Q_E_ between wet and dry patches); at the local scale (e.g., between parks, water bodies, and built-up areas of different density); and at the meso-scale (e.g., between city and surrounding rural environment: urban breeze; coastal cities sea breezes; topographic induced anabatic/katabatic effects). Moisture advection enhances latent heat flux and can be of similar size to Q_H_, but opposite in sign, thereby essentially offsetting each other^45^. For this local scale study the advection error is within the Q_F_ estimate from energy balance closure. 3) Any unmeasured terms are incorporated in the error of the Q_F_ estimate. The resulting Q_F_ is evaluated by comparison to Q_F_ estimates from alternative approaches, based on inventories^29,33,46^.

### Data availability

This research was conducted under the framework of the URBANFLUXES project that received funding from the European Union’s Horizon 2020 Research and Innovation Programme and joined the H2020 Pilot on Open Research Data. Therefore, all datasets related to the present study are included in the URBANFLUXES Data Repository as Open Data and can be accessed at: http://urbanfluxes.eu/data/.

## Electronic supplementary material

Supplementary Information
