# Supplementary material for: Urban energy exchanges monitoring from space
Source: Sci Rep. 2018 Jul 31;8:11498. doi: 10.1038/s41598-018-29873-x (PMC6068159; doi:10.1038/s41598-018-29873-x)
Supplement: Supplementary file 1 — Supplementary Information [file 41598_2018_29873_MOESM1_ESM.pdf]

Supplementary Information

Urban energy exchanges monitoring from space

Nektarios Chrysoulakis[1*], Sue Grimmond[2], Christian Feigenwinter[3], Fredrik Lindberg[4], Jean-Philippe Gastellu-Etchegorry[5], Mattia Marconcini[6], Zina Mitraka[1], Stavros Stagakis[1], Ben Crawford[2], Frans Olofson[4], Lucas Landier[5], William Morrison[2] and Eberhard Parlow[3]

[1]Foundation for Research and Technology Hellas, Institute of Applied and Computational Mathematics, N. Plastira 100, Vassilika Vouton, 70013, Heraklion, Greece, zedd2@iacm.forth.gr
[2]University of Reading, UK, c.s.grimmond@reading.ac.uk
[3]University of Basel, Switzerland, christian.feigenwinter@unibas.ch
[4]University of Gothenburg, Sweden, fredrikl@gvc.gu.se
[5]Centre d'Etude Spatiale de la Biosphère (CESBIO), France, jean-philippe.gastellu-etchegorry@cesbio.cnes.fr
[6]German Aerospace Centre (DLR), Germany, mattia.marconcini@dlr.de

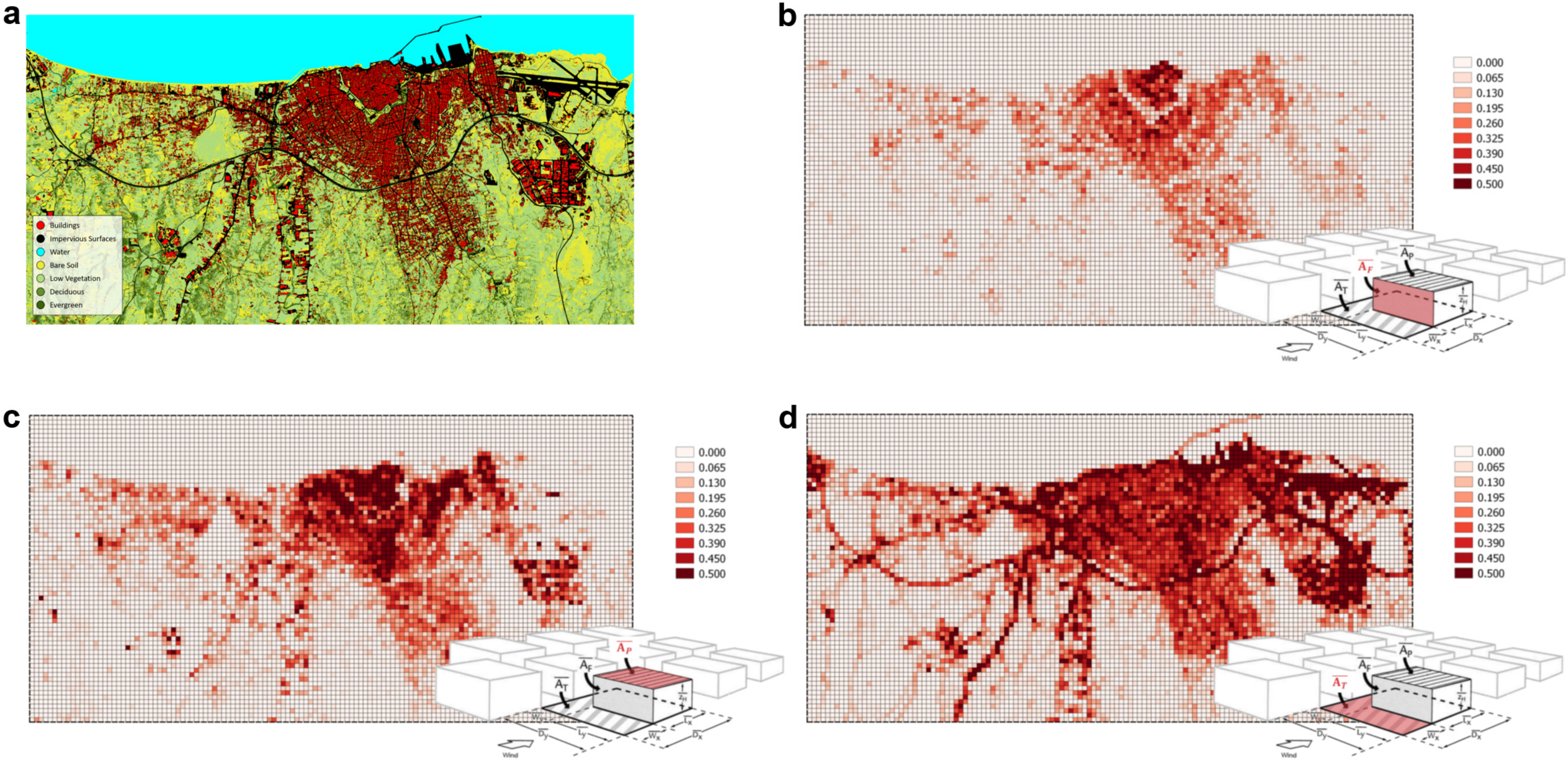

Figure S1 | Land cover map for Heraklion, Greece and corresponding fractions of morphological analysis products per each 100 m x 100 m grid cell. **(a)** Land cover map with seven information classes: buildings, paved surfaces (e.g., roads, parking lots, sidewalks), water, bare soil, low vegetation (< 2 m) and high vegetation (≥ 2 m) split into evergreen and deciduous trees. **(b)** Frontal area index. **(c)** Plan area index. **(d)** Impervious area fraction. Maps created with QGIS software, version 2.18 (www.qgis.org).

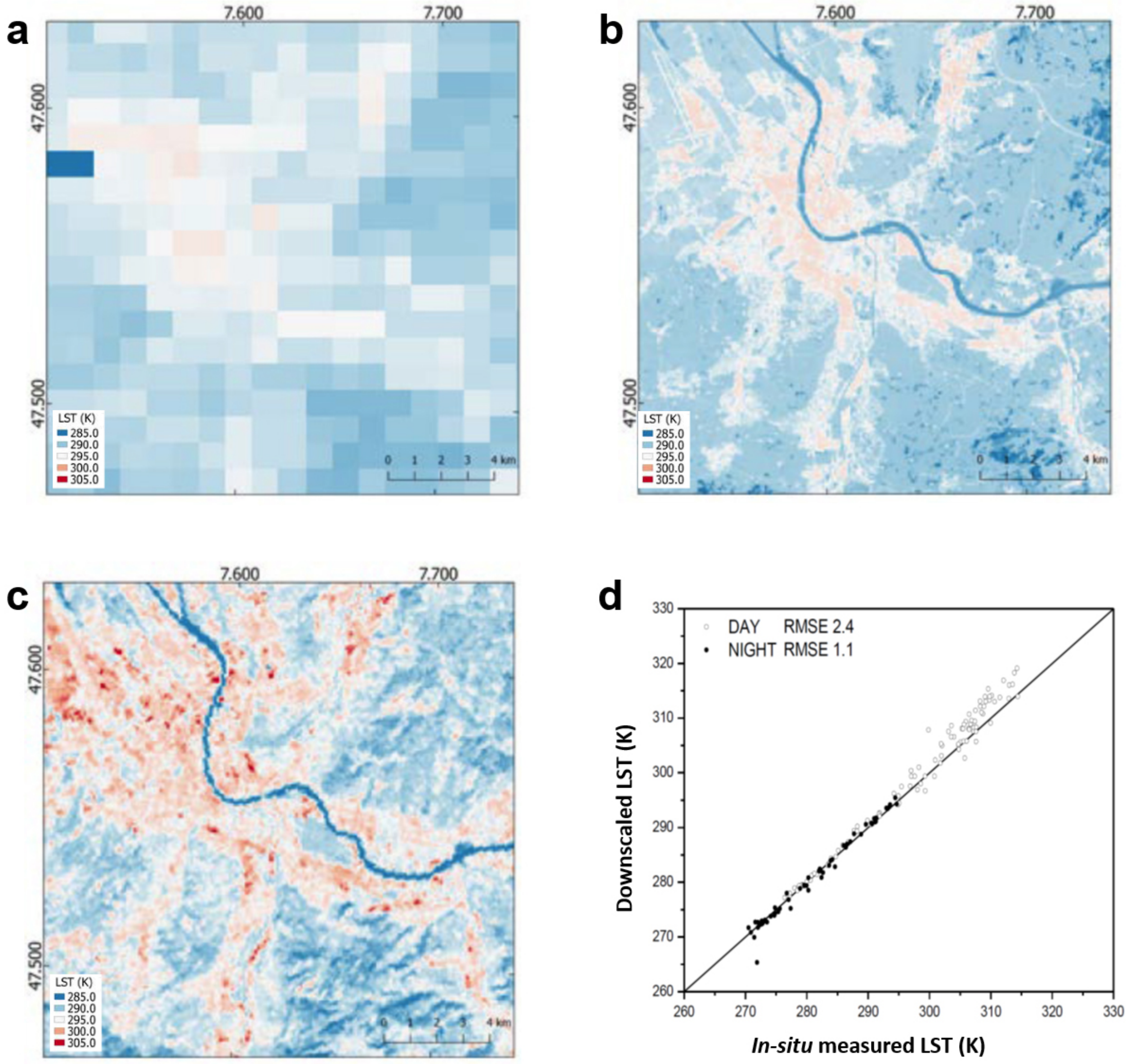

Figure S2 | Example of downscaled surface temperature for Basel, Switzerland and corresponding quality assessment. **(a)** LST as derived from MODIS thermal infrared observations at city scale (1 km x 1 km). **(b)** Downscaled surface temperature derived from MODIS observations. **(c)** LST derived from ASTER observations at local scale (90 m x 90 m). Acquisition for both MODIS and ASTER imagery: 28 March 2014 at 10:30 local time. **(d)** Evaluation of downscaled LST for 2016, using in-situ LST observations at the BLKI flux tower (see Fig. 4). Grid reference is geographical latitude/longitude WGS84 (EPSG:4326). Maps created with QGIS software, version 2.18 (www.qgis.org).

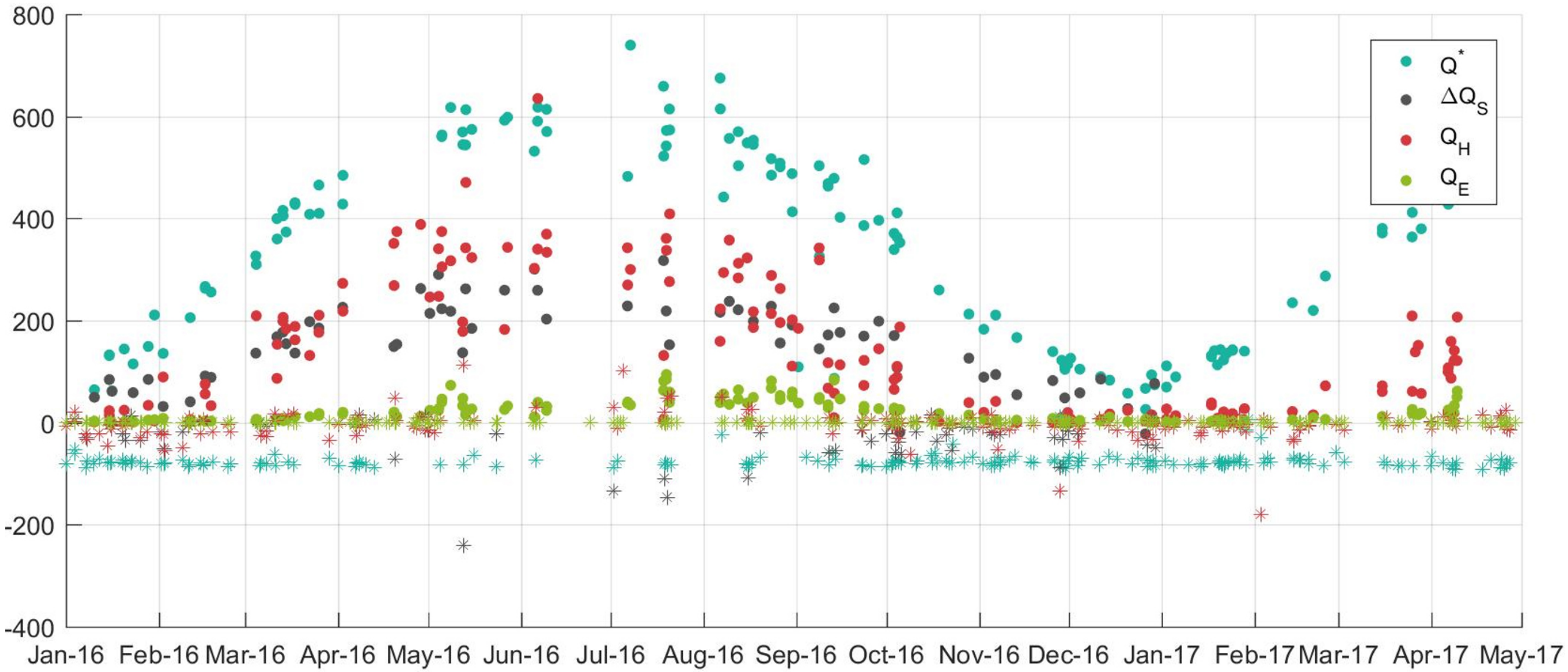

Figure S3 | Median values of UEB fluxes (EO-derived at 100 m x 100 m), for non-vegetated and non-water pixels for London for 2016 and part of 2017. The fluxes follow the expected seasonal cycle with: net all-wave radiation (Q*) peaking in summer (minima in January), net change in heat storage (ΔQ_S) and turbulent heat flux (Q_H) having similar behaviour (minima in December, maxima in August), whereas turbulent latent heat flux (Q_E) being relatively small throughout the year. Day-time (dots) and nigh-time (crosses) are shown. Note: EO requires clear skies for acquisition.

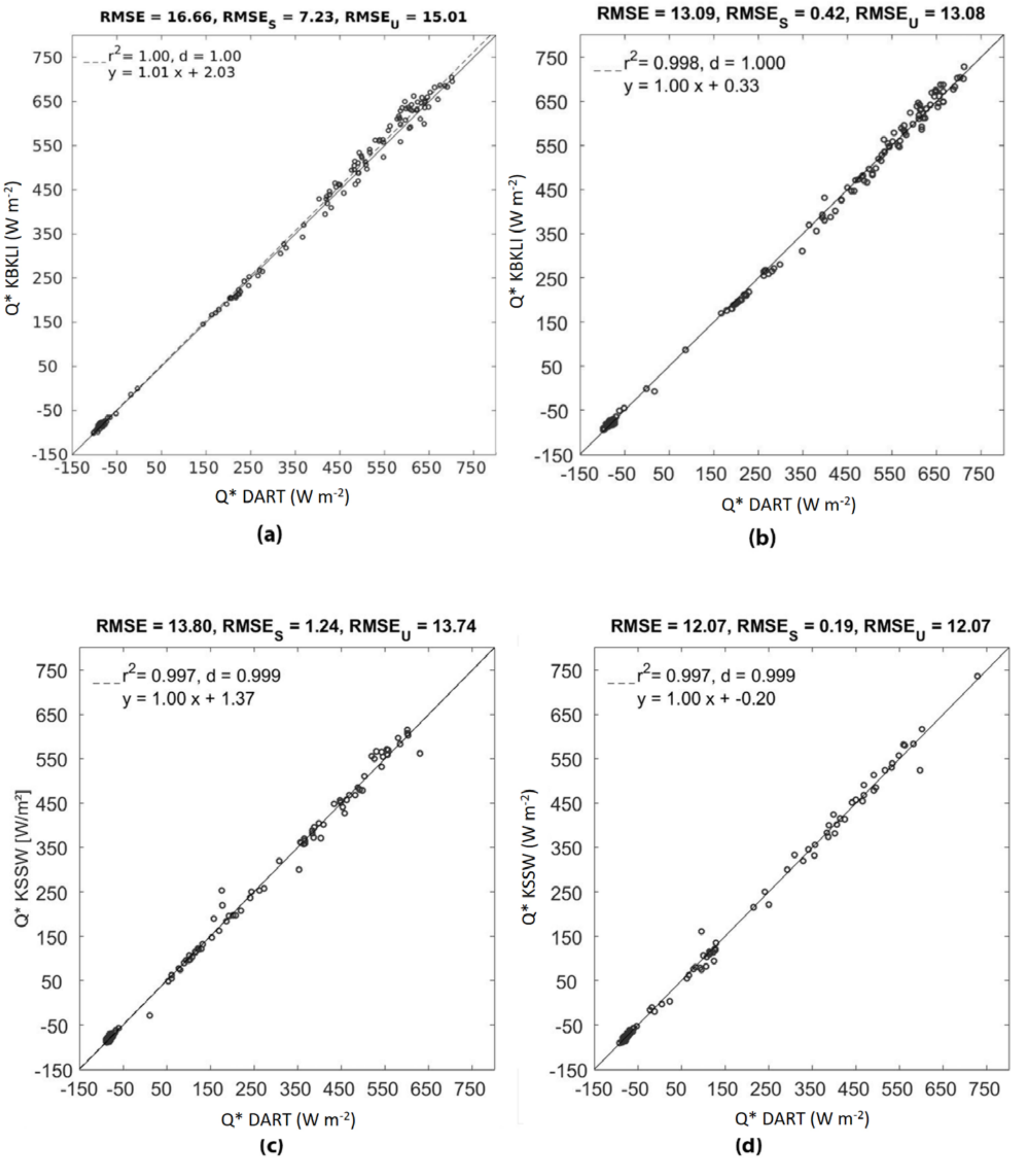

Figure S4 | Net-all wave radiation Q^* measured at flux towers versus simulated with DART using Sentinel 2 imagery, at the time of (**a, c**) MODIS/Aqua and **(b, d)** MODIS/Terra for **(a,b)** Basel (BKLI site) and (**c, d)** London (KSSW site).

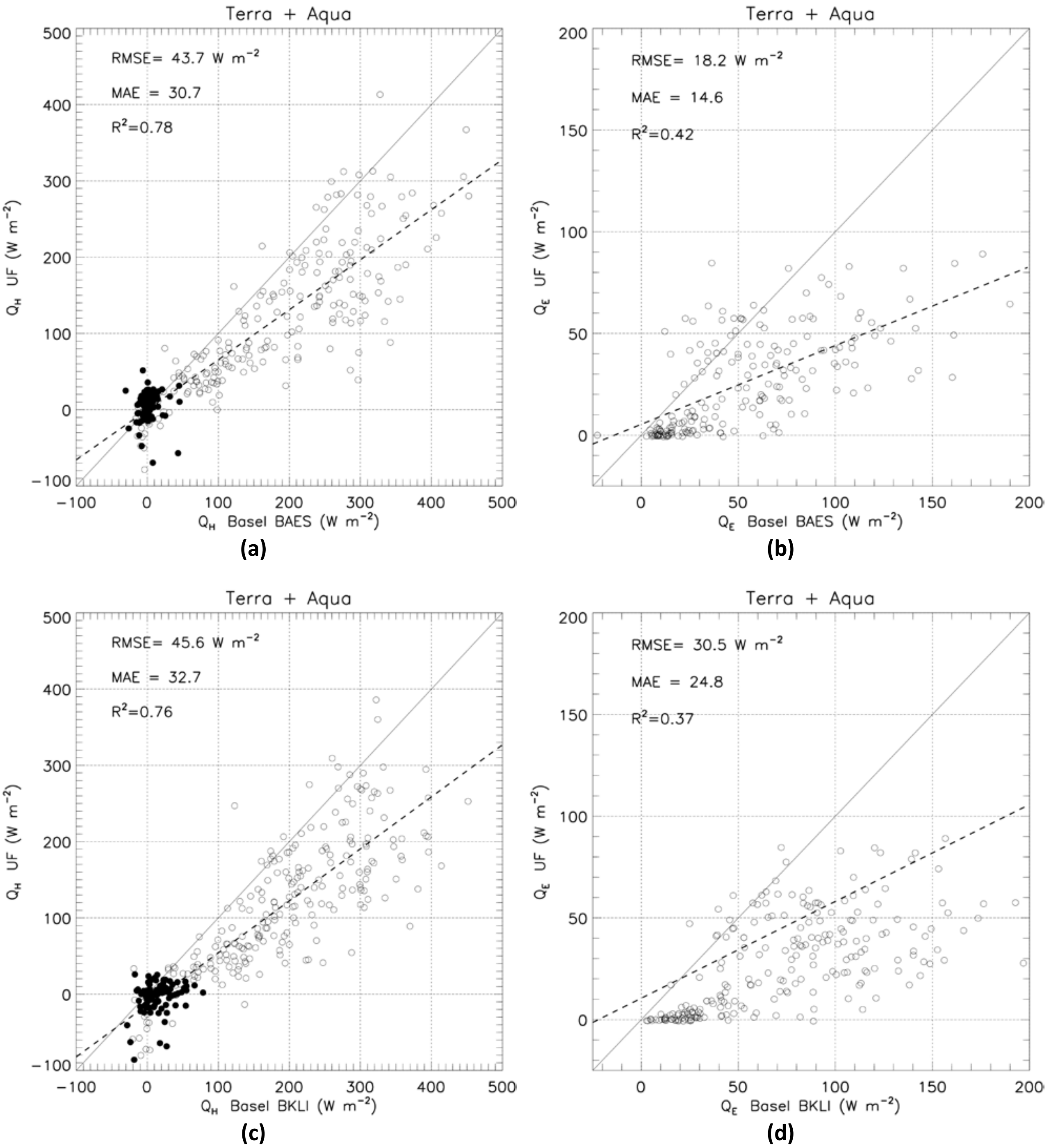

Figure S5 | Sensible (Q_H) and latent (Q_E) heat fluxes measured at Eddy Covariance towers versus satellite-based estimates for: **(a-b)** Basel (BAES site); **(c-d)** Basel (BKLI site); where (**a, c**) are Q_H ,and **(b, d)** are Q_E.

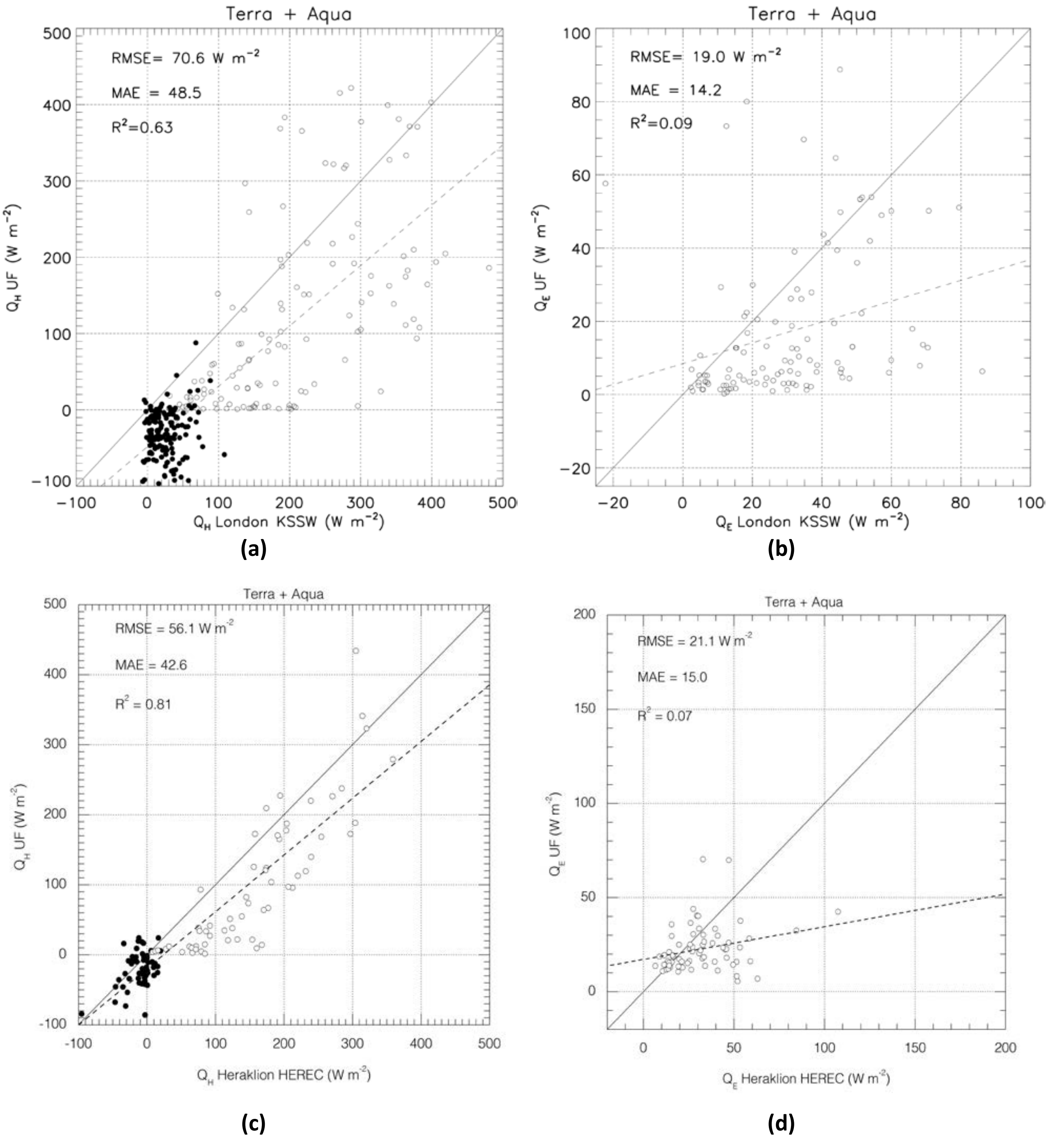

Figure S6 | Sensible (Q_H) and latent (Q_E) heat fluxes measured at Eddy Covariance towers versus satellite-based estimates for: **(a-b)** London (KSSW site) and **(c-d)** Heraklion (HEREC site); where (**a, c**) are Q_H ,and **(b, d)** are Q_E.
